# Acne treated with tumor necrosis factor inhibitors

**DOI:** 10.1016/j.jdin.2026.04.011

**Published:** 2026-04-22

**Authors:** Lia G. Bizuneh, Sarah J. Coates, Haley B. Naik, Erin Mathes, Erin Amerson

**Affiliations:** a University of California, San Francisco School of Medicine, San Francisco, California; b Department of Dermatology, Zuckerberg San Francisco General Hospital, San Francisco, California; c Department of Dermatology, University of California, San Francisco, San Francisco, California

**Keywords:** acne, acne fulminans, adalimumab, biologic therapy, follicular occlusion disorders, infliximab, pediatric acne, retrospective chart review, tumor necrosis factor inhibitors

*To the Editor:* Tumor necrosis factor-α inhibitors (TNFi) have been used off-label to treat severe or refractory acne, although evidence remains limited to case reports and small case series,^1^, ^2^, ^3^ which are subject to reporting bias. We aimed to characterize the demographic and clinical characteristics, treatment patterns, and outcomes of acne patients treated with TNFi at 2 University of California, San Francisco–affiliated centers.

We conducted a retrospective chart review of adult and pediatric patients with acne diagnosed and treated by dermatologists between 2019 and 2025 at the University of California, San Francisco and Zuckerberg San Francisco General Hospital (IRB # 20-33109). We identified patients with at least 1 International Classification of Diseases, 10th Revision code for acne and at least 1 TNFi prescription, including only those with documented follow-up and clinical description of acne response after TNFi initiation. We reported demographics, treatment regimens, prior and concurrent therapies, associated cutaneous diseases and systemic syndromes, clinical outcomes, and adverse events. Findings were summarized using descriptive statistics.

Thirty-one patients were included (Table I) (93.5% male; 35.5% White, 19.4% Hispanic/Latino, 16.1% Asian, 12.9% Black, 16.1% other). Median age at TNFi initiation was 18 years (range: 8-53 years). Indications for TNFi included acne with hidradenitis suppurativa (HS) (35.5%), acne with HS and dissecting cellulitis of the scalp (22.6%), and acne alone (22.6%). Most patients (30/31, 96.8%) received adalimumab, and 4 received infliximab, 3 of whom previously failed adalimumab (Table II). Concurrent therapies included isotretinoin (29.1%), antibiotics (35.5%), and prednisone (16.1%), with 74.2% receiving at least 1 concurrent therapy. In patients with acne alone, TNFi therapy was initiated only after intolerance and/or failure of at least 1 additional systemic therapy (eg, isotretinoin, antibiotics). Nineteen (61.3%) were treated with isotretinoin prior to TNFi initiation; 9 continued isotretinoin during TNFi therapy. Eleven patients were diagnosed with acne fulminans before TNFi initiation, 3 while taking isotretinoin.

**Table I:** Characteristics of acne patients treated with TNFi

Characteristic	All patients (*N* = 31)
Age in y, median (range)	18.0 (8-53)
≤18	17 (54.8%)
>18	14 (45.2%)
Sex	
Male	29 (93.5%)
Female	2 (6.5%)
Race/ethnicity	
White	11 (35.5%)
Black	4 (12.9%)
Asian	5 (16.1%)
Hispanic and/or Latino	6 (19.4%)
Other	5 (16.1%)
Clinical site	
UCSF	28 (90.3%)
ZSFG	3 (9.7%)
Disease	
Acne∗ alone	7 (22.6%)
Acne and HS	11 (35.5%)
HS, acne, DCS	7 (22.6%)
HS, acne, DCS, and pilonidal sinus	2 (6.5%)
Acne with DCS	1 (3.2%)
SAPHO	1 (3.2%)
PASH	1 (3.2%)
Nevus comedonicus	1 (3.2%)
TNFi dosing regimens†	
Adalimumab 40 mg every 7 d	13 (41.9%)
Adalimumab 40 mg every 14 d	6 (19.4%)
Adalimumab 80 mg every 7 d	4 (12.9%)
Adalimumab 80 mg every 14 d	5 (16.1%)
Adalimumab 20 mg every 14 d	1 (3.2%)
Infliximab 7.5 mg/kg every 6 wk	1 (3.2%)
Infliximab 12 mg/kg every 4 wk	1 (3.2%)
Infliximab 12.5 mg/kg every 4 wk	2 (6.5%)
Coadministered therapies‡	
Isotretinoin	9 (29.0%)
Antibiotics§	8 (25.8%)
Prednisone	5 (16.1%)
Dapsone	3 (9.7%)
Tofacitinib	1 (3.2%)
Topical therapy‖	13 (41.9%)

*DCS*, Dissecting cellulitis of the scalp; *HS*, hidradenitis suppurativa; *PASH*, pyoderma gangrenosum, acne, and suppurative hidradenitis; *SAPHO*, synovitis, acne, pustulosis palmaris, hyperostosis, and osteitis; *TNFi*, tumor necrosis factor-α inhibitors; *UCSF*, University of California, San Francisco; *ZSFG*, Zuckerberg San Francisco General Hospital.

∗ Definition of acne includes acne fulminans, nodulocystic acne, acne conglobata, acne cystica, and acne vulgaris. All diagnoses were established by board-certified dermatologists.

† Most recent regimen to date.

‡ Includes therapies coadministered with adalimumab (*n* = 30) and/or infliximab (*n* = 4). Some patients received both agents sequentially. 58% of systemic therapies were administered prior to TNFi initiation, 19% initiated after.

§ Antibiotics administered include: Clindamycin, Rifampin, Trimethoprim / Sulfamethoxazole, Doxycycline, Ertapenem, and Cefadroxil.

‖ Topical therapies include: retinoids (tretinoin or adapalene), benzoyl peroxide, topical clindamycin, topical dapsone, and clobetasol.

**Table II:** Clinical outcomes of acne patients treated with TNF inhibitors

Outcome	TNFi agent			By diagnosis∗		By systemic therapy	
	Adalimumab (*n* = 30)	Infliximab (*n* = 4)	All TNFi† (*N* = 31)	Acne only (*n* = 7)	Acne + follicular occlusion disorder‡ (*n* = 21)	TNFi alone (*n* = 7)	TNFi + systemic (*n* = 24)
TNFi treatment response§							
Clinical improvement (any)	26 (86.7%)	2 (50%)	28 (90.3%)	7 (100%)	18 (85.7%)	5 (71.4%)	22 (91.7%)
No acne improvement	4 (13.3%)	2 (50.0%)	3 (9.7%)	0 (0.0%)	3 (14.2%)	2 (28.6%)	2 (8.3%)
Partial acne improvement	17 (56.7%)	2 (50%)	19 (61.3%)	5 (71.4%)	12 (57.1%)	4 (57.1%)	14 (58.3%)
Significant acne improvement	9 (30%)	0 (0.0%)	9 (29.0%)	2 (28.6%)	6 (28.6%)	1 (14.3%)	8 (33.3%)
Discontinued due to adverse effect‖	4 (13.3%)	0 (0.0%)	4 (12.9%)	2 (28.6%)	2 (4.3%)	0 (0.0%)	4 (16.7%)
Time to improvement in mo, median (IQR)	1.5 (0.6-3.0)	N/A	1.5 (0.6-3.0)	1.5 (0.6-2.8)	1.5 (0.8-3.0)	3.0 (1.8-3.5)	1.0 (0.7-3.0)
Clinical improvement by diagnosis							
Comorbid condition clinical improvement¶	13 (43.3%)	2 (50%)	15 (48.4%)	N/A	16 (76.2%)	4 (57.1%)	11 (45.8%)
Acne fulminans clinical improvement	N/A	N/A	11 (100%)	6 (100%)	5 (100%)	1 (100%)	10 (100%)

*IQR*, Interquartile range; *N/A*, not applicable; *TNFi*, tumor necrosis factor-α inhibitors.

∗ Nevus comedonicus, PASH, and SAPHO (*n* = 3) excluded from “by diagnosis” section of table; all 3 demonstrated clinical improvement on adalimumab (2 partial, 1 significant).

† Patients may appear under both Adalimumab and Infliximab columns if they received >1 TNFi. “All TNFi” column, each patient is counted once (*N* = 31), outcomes are attributed to the latest TNFi treatment received. Three patients switched from adalimumab to infliximab; 1 partially improved; 2 no response. There was no clear relationship between TNFi dose/frequency and acne response.

‡ Includes acne + HS, acne + DCS, follicular occlusion triad and tetrad.

§ Clinical improvement was defined as provider-documented improvement in acne after TNFi initiation. We designated ‘partial’ improvement if the following keywords were found in the chart in relation to acne response post TNFi treatment: “some”, “partial”, “improving”, “improved”, and “significant” improvement if the following keywords were found: “significant”, “improved dramatically”, “much improved”, and “clear”. Acne recurrence after TNFi discontinuation: 6/31 (19.4%).

‖ Adverse effects include psoriasis flare, psoriasiform rash, drug-induced arthralgias, and LFT elevation. All were on TNFi long enough to determine treatment response before discontinuation. Two were counted toward significant improvement, 1 partial improvement, and 1 no improvement.

¶ Comorbid improvement defined as provider-documented improvement in the concomitant condition (HS, DCS, or syndromic disease). Of note, 82.5% of acne responders also had improvement in comorbid conditions, while all acne nonresponders also had no response of comorbid conditions (eg, HS, DCS).

Acne improved in 90.3% of patients. Median time to improvement was 2 months with adalimumab (interquartile range [IQR]: 1.0-3.4) and 1 month with infliximab (IQR: 1.0-1.0). Median TNFi duration was 10 months (IQR, 5-19 months; range, 1.5-28 months). While most patients continued additional acne therapies, improvement was temporally associated with TNFi initiation. 8/9 with significant acne improvement did not use isotretinoin concurrently, nor did they have isotretinoin-induced acne fulminans. Four (12.9%) discontinued TNFi due to adverse effects (Table II).

These results suggest that TNFi may be effective for severe or refractory acne. A 2023 systematic review aggregated 17 reported cases of nonsyndromic (eg, non–synovitis, acne, pustulosis palmaris, hyperostosis, and osteitis, pyoderma gangrenosum, acne, and suppurative hidradenitis) acne.^1^ They similarly observed male predominance, high rates of clinical response, and rapid improvement. Compared with prior reports,^1^, ^2^, ^3^ our cohort disproportionately had at least 1 other follicular occlusion disorder (eg, HS, dissecting cellulitis of the scalp), likely reflecting prescribing practices and insurance coverage for these comorbidities.

Limitations include retrospective design, variable follow-up duration, concurrent therapies limiting attribution of improvement to TNFi alone, and reliance on keyword identification of “partial” and “significant” in provider documentation rather than standardized acne severity scores^4^ or nodule counts.

Prospective studies are needed to establish efficacy, safety, and optimal use of TNFi for managing severe or refractory acne.

## Conflicts of interest

None disclosed.
